# Does Combination Therapy With a Glucagon-Like Peptide-1 Receptor Agonist and a Sodium-Glucose Cotransporter 2 Inhibitor Enhance Cardiovascular and Renal Protection in People With Type 2 Diabetes Mellitus?

**DOI:** 10.7759/cureus.112724

**Published:** 2026-07-15

**Authors:** Regina Medeiros, Inês Mendes

**Affiliations:** 1 Endocrinology and Nutrition, Hospital Divino Espírito Santo, Ponta Delgada, PRT

**Keywords:** chronic kidney disease, glucagon‐like peptide‐1 receptor agonists, heart failure, major adverse cardiovascular events, sodium-glucose cotransporter 2 inhibitors

## Abstract

The glucagon-like peptide-1 receptor agonist (GLP-1 RA) plus sodium-glucose cotransporter 2 inhibitor (SGLT2i) group shows a lower risk of major adverse cardiovascular events (MACE), admission due to heart failure (HF), and worsening chronic kidney disease (CKD), as determined by exploratory analysis; however, statistical superiority criteria were not met.

The major cause of mortality in patients with diabetes is cardiovascular disease, and it is crucial to address this burden. This study evaluated the cardiovascular effects of combining glucagon-like peptide-1 receptor agonists (GLP-1 RAs) with sodium-glucose cotransporter 2 inhibitors (SGLT2is) versus either drug alone in patients with type 2 diabetes (T2D).

This retrospective cohort study used data from 238 patients with a diagnosis of T2D aged 18 years or older who were followed for at least four years by the Endocrinology Department at Hospital Divino Espírito Santo, Ponta Delgada, Portugal. We included individuals who were treated for at least one year with a combination of GLP-1 RA and SGLT2i (n=99), compared with GLP-1 RA monotherapy (n=61) and SGLT2i monotherapy (n=78). The primary outcome was the reduction of major adverse cardiovascular events. The secondary outcomes were the reduction of worsening chronic kidney disease, the reduction of admissions due to heart failure, and the safety profile.

## Introduction

Diabetes contributes to the development of cardiovascular disease, renal disease, and other complications, resulting in significant economic burdens for both patients and the healthcare system and substantially diminishing patients’ quality of life.

Optimized glycemic control is a key factor in avoiding the onset and progression of diabetes-related complications. Recent pharmacotherapeutic advancements have consequently shifted management strategies for type 2 diabetes (T2D).

The introduction of glucagon-like peptide-1 receptor agonists (GLP-1 RAs) and sodium-glucose cotransporter 2 inhibitors (SGLT2is) has transformed the management paradigm for T2D. These drug classes not only effectively control glucose levels but also provide cardiorenal protective effects independent of glycemic control, including lowering blood pressure and body weight, reducing inflammation, and improving glomerular, endothelial, and cardiac function [1-10].

This study evaluated whether combination therapy with GLP-1 RAs and SGLT2is offers greater cardiovascular protection than SGLT2i or GLP-1 RA monotherapy in patients with T2D in a real-world setting.

## Materials and methods

This is a retrospective cohort study. The present study focused on patients who met the following criteria: aged 18 years or older, diagnosed with T2D, and followed by the Department of Endocrinology at the Hospital Divino Espírito Santo, Ponta Delgada, Portugal, for at least four years. Data were collected from April 1, 2022, to January 31, 2025, with an initial three cohorts established: those receiving both GLP-1 RAs and SGLT2is (n=99), those receiving only SGLT2is (n=78), and those receiving only GLP-1 RAs (n=61).

The exclusion criteria were the presence of malignancy, lack of adherence to treatment, follow-up for less than four years, concomitant use of glucocorticoids, and uncertain diabetes classification.

We collected demographic/clinical data, baseline comorbidities, medications, and laboratory values obtained by consulting each patient’s medical record. Demographics included sex, age, race, initial and final body mass index (BMI), and duration of T2D. Comorbidities included ischemic heart disease, peripheral vascular disease, cerebrovascular disease, heart failure (HF), and cardiovascular risk factors without established cardiovascular disease (hypertension and dyslipidemia). Medications included SGLT2is, GLP-1 RAs, other antidiabetic agents, antihypertensives, lipid-lowering agents, and antithrombotics. Laboratory measures included glycated hemoglobin (HbA1c) and estimated glomerular filtration rate (eGFR).

The primary outcome was the occurrence of major adverse cardiovascular events (MACE), including acute myocardial infarction, ischemic stroke, transient ischemic attack, acute limb ischemia, cardiac arrest, and cardiovascular death. The secondary outcomes were worsening chronic kidney disease (CKD) and hospitalization due to HF.

Statistical analysis was performed using KNIME Analytics Platform® version 5.3.1 for Windows (KNIME GmbH, Körtestr, Berlin), with R integration. To assess the normality of the numerical variables, we used the Kolmogorov-Smirnov test, which indicated that none followed a normal distribution. Therefore, continuous numerical variables were reported as median (interquartile range (IQR)), while categorical variables were reported as number (%). To compare categorical variables, we used the chi-square test.

Time-to-event analyses were performed for cardiovascular mortality, heart failure hospitalization, and CKD progression. Kaplan-Meier survival curves were generated for each treatment group and compared using the log-rank test. Follow-up time was defined as the interval between treatment initiation and the occurrence of the event (MACE, hospitalization due to HF, or worsening CKD) or the end of the study (January 2025), whichever occurred first. Multivariable Cox proportional hazards regression models were used to estimate hazard ratios (HRs) and 95% confidence intervals (CIs), adjusting for clinically relevant baseline covariates. For cardiovascular mortality and heart failure hospitalization, the models included age, sex, baseline presence of HF, BMI, and insulin use. For CKD progression, the models included age, baseline CKD stage, and baseline BMI.

To further reduce confounding due to baseline differences between treatment groups, inverse probability of treatment weighting (IPTW) based on propensity scores was applied. Propensity score weights were estimated using multinomial logistic regression including age, sex, baseline presence of heart failure (or baseline CKD stage for the CKD analysis), baseline BMI, and insulin use. Covariate balance after weighting was assessed using standardized mean differences. IPTW-weighted Cox proportional hazards models with robust standard errors were then fitted to estimate adjusted hazard ratios and corresponding 95% confidence intervals. The proportional hazards assumption was assessed using Schoenfeld residuals for both multivariable and IPTW-weighted Cox models.

A two-sided p-value of <0.05 was considered statistically significant.

## Results

Table 1 summarizes the baseline characteristics of the study population.

**Table 1:** Baseline characteristics of the study population ACEi: angiotensin-converting enzyme inhibitors, ARB: angiotensin receptor blockers, CCB: calcium channel blockers, eGFR: estimated glomerular filtration rate, GLP-1 RA: glucagon-like peptide-1 receptor agonist, HbA1c: glycated hemoglobin, IQR: interquartile range, T2D: type 2 diabetes mellitus, SGLT2i: sodium-glucose cotransporter 2 inhibitors

Parameters	SGLT2i (n=78)	GLP-1 RA (n=61)	SGLT2i + GLP-1 RA (n=99)
Sex (female), number (%)	40 (51.3)	50 (82.0)	75 (75.8)
Age (year), median (IQR)	71.0 (66.0-73.0)	64.0 (56.0-69.0)	65.0 (59.0-70.0)
Race (Caucasian), number (%)	78 (100.0)	61 (100.0)	99 (100.0)
Initial body mass index (kg/m^2^), median (IQR)	30.3 (27.4-34.1)	33.1 (29.8-38.3)	33.8 (31.1-37.2)
Final body mass index (kg/m^2^), median (IQR)	30.2 (27.1-34.1)	32.0 (28.1-37.2)	32.8 (29.2-35.8)
T2D duration			
Between 1 and 5 years, number (%)	1 (1.3)	1 (1.6)	2 (2.0)
Between 5 and 10 years, number (%)	15 (19.2)	17 (27.9)	24 (24.2)
More than 10 years, number (%)	62 (79.5)	43 (70.5)	73 (73.7)
Comorbidities			
Ischemic heart disease, number (%)	16 (20.5)	11 (18.0)	14 (14.1)
Peripheral vascular disease, number (%)	17 (21.8)	11 (18.0)	16 (16.2)
Cerebral vascular disease, number (%)	10 (12.8)	5 (8.2)	9 (9.1)
Heart failure, number (%)	11 (14.1)	4 (6.6)	13 (13.1)
Hypertension, number (%)	74 (94.9)	49 (80.3)	84 (84.8)
Dyslipidemia, number (%)	67 (85.9)	51 (83.6)	80 (80.8)
Medication			
SGLT2 inhibitor			
Dapagliflozin, number (%)	54 (69.2)	-	78 (78.0)
Empagliflozin, number (%)	20 (25.6)	-	16 (16.0)
Canagliflozin, number (%)	3 (3.8)	-	5 (5.0)
Ertugliflozin, number (%)	1 (1.3)	-	1 (1.0)
Total time under iSGLT2i (years), median (IQR)	5.0 (3.0-9.0)	-	7.0 (4.0-8.0)
Total time under iSGLT2i during the study time (years), median (IQR)	4.0 (3.0-4.0)	-	4.0 (3.0-4.0)
GLP-1 RA			
Dulaglutide, number (%)	-	25 (41.0)	51 (51.0)
Semaglutide subcutaneous, number (%)	-	17 (27.9)	16 (16.0)
Liraglutide, number (%)	-	9 (14.8)	7 (7.0)
Exenatide, number (%)	-	10 (16.4)	26 (26.3)
Total time under GLP-1 RA (years), median (IQR)	-	4.0 (2.0-6.0)	5.0 (2.0-8.0)
Total time under GLP-1 RA during the study time (years), median (IQR)	-	4.0 (2.0-4.0)	4.0 (2.0-4.0)
Other hypoglycemic drugs			
None, number (%)	0 (0)	1 (1.6)	2 (2.0)
Metformin, number (%)	62 (79.5)	41 (67.2)	78 (78.8)
DPP4 inhibitors, number (%)	21 (26.9)	18 (29.5)	35 (35.4)
Sulfonylureas, number (%)	12 (15.4)	11 (18.0)	14 (14.1)
Pioglitazone, number (%)	2 (2.6)	2 (3.3)	3 (3.0)
Insulin, number (%)	54 (69.2)	46 (75.4)	71 (71.7)
Antihypertensives drugs			
None, number (%)	4 (5.1)	12 (19.7)	15 (15.2)
ECAi/ARB, number (%)	69 (88.5)	46 (75.4)	72 (72.7)
Diuretic, number (%)	37 (47.5)	30 (49.2)	60 (60.6)
CCB, number (%)	38 (48.7)	21 (34.4)	42 (42.4)
Beta-blocker, number (%)	23 (29.5)	14 (23.0)	30 (30.3)
Alpha-blocker, number (%)	6 (7.7)	3 (4.9)	17 (17.2)
Lipid-lowering drugs			
None, number (%)	11 (14.1)	10 (16.4)	19 (19.2)
Statin, number (%)	43 (55.1)	28 (45.9)	50 (50.5)
Fibrates, number (%)	34 (30.8)	23 (37.7)	30 (30.3)
Antithrombotic drugs			
None, number (%)	39 (50.0)	42 (68.8)	66 (66.7)
Antiplatelet, number (%)	32 (41.0)	14 (23.0)	29 (29.3)
Anticoagulation, number (%)	7 (9.0)	6 (8.2)	4 (4.0)
Laboratory			
HbA1c, %, median (IQR)	7.3 (6.6-8.0)	7.4 (6.4-8.2)	7.8 (7.2-8.4)
Initial eGFR (1.73 mL/min.1.73 m^2^)			
>90, number (%)	17 (21.8)	20 (32.8)	28 (28.3)
60-89, number (%)	39 (50.0)	26 (42.6)	45 (45.4)
30-59, number (%)	17 (21.8)	13 (21.3)	22 (22.2)
29-15, number (%)	2 (2.6)	1 (1.6)	4 (4.0)
15-0, number (%)	0 (0.0)	0 (0.0)	0 (0.0)
Final eGFR (1.73 mL/min.1.73 m^2^)			
>90, number (%)	18 (23.1)	17 (27.9)	26 (26.0)
60-89, number (%)	30 (38.5)	23 (37.7)	41 (41.0)
30-59, number (%)	21 (26.9)	11 (18.0)	24 (24.0)
29-15, number (%)	3 (3.8)	4 (6.6)	3 (3.0)
15-0, number (%)	0 (0.0)	0 (0.0)	0 (0.0)

The average age was 65 (59-70) years for the GLP-1 RA plus SGLT2i group, 71 (66-73) years for the SGLT2i-only group, and 64 (56-69) years for the GLP-1 RA-only group.

The GLP-1 RA plus SGLT2i group had a median initial BMI of 33.8 (31.1-37.2) kg/m² and an age of 65.0 (59.0-70.0) years; 75.8% were women. The SGLT2i-only group had a median initial BMI of 30.3 (27.4-34.1) kg/m² and an age of 71.0 (66.0-73.0) years; 51.3% were women. The GLP-1 RA-only group had a median initial BMI of 33.1 (29.8-38.3) kg/m² and an age of 64.0 (56.0-69.0) years; 80.2% were women.

Cardiovascular risk factors, such as obesity, hypertension, and dyslipidemia, were prevalent. Regarding medication use, the percentage of individuals using insulin (74.4%) was higher in the GLP-1 RA monotherapy group than in the SGLT2i monotherapy group (69.2%) or the GLP-1 RA plus SGLT2i group (71.7%). HbA1c and eGFR showed slight differences among the three groups.

Table 2 shows that the group receiving dual therapy with GLP-1 RA and SGLT2i had a lower percentage of MACE (2.0%) than those treated with SGLT2i monotherapy (3.8%) or GLP-1 RA monotherapy (8.2%), although this difference did not reach statistical significance (p>0.05).

**Table 2:** Risk for clinical outcomes The chi-square test was used to compare categorical variables. CKD: chronic kidney disease, GLP-1 RA: glucagon-like peptide-1 receptor agonist, SGLT2i: sodium-glucose cotransporter 2 inhibitors

Clinical outcomes (events/number at risk (%))	SGLT2i (n=78)	GLP-1 RA (n=61)	SGLT2i + GLP-1 RA (n=99)	SGLT2i + GLP-1 RA versus SGLT2i (chi-square value - χ²)	SGLT2i + GLP-1 RA versus SGLT2i (p-value)	SGLT2i + GLP-1 RA versus GLP-1 RA (chi-square value - χ²)	SGLT2i + GLP-1 RA versus GLP-1 RA (p-value)
Cardiac arrest/cardiovascular death	3 (3.8)	5 (8.2)	2 (2.0)	χ²=0.547	p=0.459	χ²=3.498	p=0.061
Acute myocardial infarction	2 (2.6)	1 (1.6)	1 (1.0)	χ²=0.647	p=0.421	χ²=0.126	p=0.722
Ischemic stroke/transient ischemic attack	0 (0.0)	1 (1.6)	2 (2.0)	χ²=0.701	p=0.402	χ²=0.027	p=0.870
Acute limb ischemia	5 (6.4)	5 (8.2)	4 (4.0)	χ²=0.530	p=0.466	χ²=1.264	p=0.261
Heart failure (hospitalization)	4 (5.1)	2 (3.3)	1 (1.0)	χ²=1.316	p=0.251	χ²=1.072	p=0.300
Worsening CKD	4 (5.1)	6 (9.8)	4 (4.0)	χ²=0.130	p=0.718	χ²=2.215	p=0.137

**Figure 1:**
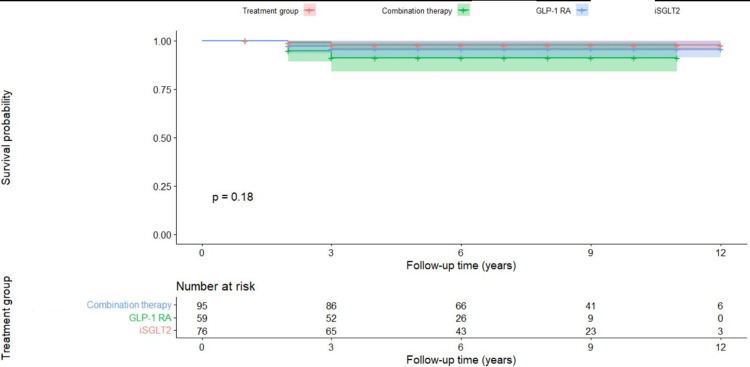
Kaplan-Meier curves for cardiovascular mortality according to treatment group GLP-1 RA: glucagon-like peptide-1 receptor agonist, SGLT2i: sodium-glucose cotransporter 2 inhibitor

During follow-up, 10 cardiovascular deaths occurred in the treated cohort: 2 in the combination therapy group, 3 in the SGLT2i group, and 5 in the GLP-1 RA group. The corresponding incidence rates were 2.96, 6.19, and 16.18 cardiovascular deaths per 1,000 person-years, respectively (Table 3). Kaplan-Meier analysis did not show significant differences in cardiovascular mortality-free survival among the SGLT2i, GLP-1 RA, and combination therapy groups (log-rank χ²=3.5, df=2, p=0.18) (Figure 1).

**Table 3:** Cardiovascular mortality rates and Cox regression analyses in treated patients Multivariable Cox model adjusted for age, sex, baseline heart failure, BMI, and insulin use. IPTW model weighted for age, sex, baseline heart failure, BMI, and insulin use, according to the propensity score model. Reference group: combination therapy (SGLT2i + GLP-1 RA). *p<0.05. HR: hazard ratio, CI: confidence interval, IPTW: inverse probability of treatment weighting, BMI: body mass index, GLP-1 RA: glucagon-like peptide-1 receptor agonist, SGLT2i: sodium-glucose cotransporter 2 inhibitor

Incidence rates of cardiovascular death			
Treatment group	Cardiovascular deaths, number	Person-years	Incidence rate per 1,000 person-years
SGLT2i	2	676	2.96
GLP-1 RA	3	485	6.19
SGLT2i + GLP-1 RA	5	309	16.18
Multivariable and IPTW-weighted Cox models for cardiovascular death			
Variable - multivariable Cox model	HR	95% CI	p-value
iSGLT2i versus SGLT2i + GLP-1 RA	1.72	0.27-11.02	0.567
GLP-1 RA versus SGLT2i + GLP-1 RA	5.31	0.96-29.27	0.056
Age	1.05	0.97-1.14	0.267
Sex	1.75	0.37-8.34	0.480
Baseline heart failure	1.72	0.35-8.56	0.509
BMI (kg/m²)	1.04	0.94-1.16	0.445
Insulin use	3.24	0.40-26.07	0.270
IPTW-weighted Cox model			
iSGLT2i versus SGLT2i + GLP-1 RA	2.92	0.40-21.17	0.290
GLP-1 RA versus SGLT2i + GLP-1 RA	7.04	1.35-36.71	0.021*

In the multivariable Cox proportional hazards model adjusted for age, sex, baseline heart failure, baseline BMI, and insulin use, no statistically significant differences were observed between treatment groups, although GLP-1 RA monotherapy showed a borderline higher hazard of cardiovascular death compared with combination therapy (HR: 5.31, 95% CI: 0.96-29.27; p=0.056), whereas SGLT2i monotherapy was not associated with a significantly different hazard (HR: 1.72, 95% CI: 0.27-11.02; p=0.567). In the IPTW-weighted Cox model, GLP-1 RA monotherapy was associated with a higher estimated hazard of cardiovascular death compared with combination therapy (HR: 7.04, 95% CI: 1.35-36.71; p=0.021), while no significant difference was observed for SGLT2i monotherapy (HR: 2.92, 95% CI: 0.40-21.17; p=0.290) (Table 3). The proportional hazards assumption was not violated in either the multivariable Cox model (global Schoenfeld test p=0.725) or the IPTW-weighted Cox model (global p=0.168). Given the low number of cardiovascular deaths and the wide confidence intervals, these findings should be interpreted cautiously.

Kaplan-Meier analysis demonstrated no significant differences in heart failure hospitalization-free survival among the treatment groups (log-rank χ²=1.8, df=2; p=0.40). During follow-up, 9 patients experienced heart failure hospitalization in the treated cohort: 2 in the combination therapy group, 4 in the SGLT2i group, and 3 in the GLP-1 RA group. The corresponding incidence rates were 2.97, 8.35, and 9.80 events per 1,000 person-years, respectively (Table 4). In the multivariable Cox model, compared with combination therapy, treatment with SGLT2i monotherapy (HR: 4.78, 95% CI: 1.28-17.91; p=0.020) and GLP-1 RA monotherapy (HR: 4.65, 95% CI: 1.16-18.72; p=0.030) was associated with a higher hazard of heart failure hospitalization. Baseline heart failure was also an independent predictor of hospitalization (HR: 27.23, 95% CI: 5.61-132.22; p<0.001). However, after IPTW, these associations were attenuated and no longer statistically significant (SGLT2i: HR: 4.09, 95% CI: 0.70-23.90; p=0.117; GLP-1 receptor agonists: HR: 6.58, 95% CI: 0.95-45.54; p=0.056) (Table 4).

**Table 4:** Heart failure hospitalization multivariable and IPTW-weighted Cox regression analyses in treated patients Multivariable Cox model adjusted for age, sex, baseline heart failure, BMI, and insulin use. IPTW model weighted for age, sex, baseline heart failure, BMI, and insulin use, according to the propensity score model. Reference group: combination therapy (SGLT2i + GLP-1 RA). HR: hazard ratio, CI: confidence interval, IPTW: inverse probability of treatment weighting, GLP-1 RA: glucagon-like peptide-1 receptor agonist, SGLT2i: sodium-glucose cotransporter 2 inhibitor, BMI: body mass index

Incidence rates of heart failure hospitalization			
Treatment group	Events, number	Person-years	Incidence rate per 1,000 person-years
SGLT2I	4	479	8.35
GLP-1 RA	3	306	9.80
SGLT2I + GLP-1 RA	2	673	2.97
Multivariable and IPTW-weighted Cox models for heart failure hospitalization			
Unadjusted log-rank	HR	95% CI	p-value
Overall group comparison	-	-	0.40
Variable - multivariable Cox model	HR	95% CI	p-value
iSGLT2I versus SGLT2i + GLP-1 RA	4.78	1.28-17.91	0.020
GLP-1 RA versus SGLT2i + GLP-1 RA	4.65	1.16-18.72	0.030
IPTW-weighted Cox model			
iSGLT2I versus SGLT2i + GLP-1 RA	4.09	0.70-23.90	0.117
GLP-1 RA versus SGLT2i + GLP-1 RA	6.58	0.95-45.54	0.056

During follow-up, 14 patients experienced CKD progression: 4 in the combination therapy group, 4 in the SGLT2i group, and 6 in the GLP-1 RA group. The corresponding incidence rates were 6.03, 8.37, and 20.3 events per 1,000 person-years, respectively (Table 5). Kaplan-Meier analysis demonstrated no significant differences in CKD progression-free survival among the three treatment groups (log-rank p=0.30). In the multivariable Cox proportional hazards model, neither SGLT2i monotherapy (HR: 1.34, 95% CI: 0.31-5.88; p=0.698) nor GLP-1 RA monotherapy (HR: 3.14, 95% CI: 0.87-11.37; p=0.082) was significantly associated with CKD progression compared with combination therapy. Similarly, after inverse probability of treatment weighting (IPTW) to account for baseline differences between treatment groups, no statistically significant associations were observed for SGLT2i monotherapy (HR: 1.56, 95% CI: 0.34-7.21; p=0.572) or GLP-1 RA monotherapy (HR: 2.89, 95% CI: 0.79-10.57; p=0.109) relative to combination therapy (Table 5).

**Table 5:** Chronic kidney disease progression multivariable and IPTW-weighted Cox regression analyses in treated patients Multivariable Cox model adjusted for age, baseline CKD stage, and baseline BMI. IPTW model weighted for age, sex, baseline CKD stage, baseline BMI, and insulin use, according to the propensity score model. Reference group: combination therapy (SGLT2i + GLP-1 RA). *p<0.05. HR: hazard ratio, CI: confidence interval, IPTW: inverse probability of treatment weighting, GLP-1 RA: glucagon-like peptide-1 receptor agonist, SGLT2i: sodium-glucose cotransporter 2 inhibitor, CKD: chronic kidney disease

Incidence rates of chronic kidney disease progression			
Treatment group	Events, number	Person-years	Incidence rate per 1,000 person-years
SGLT2i	4	478	8.37
GLP-1 RA	6	296	20.3
SGLT2i + GLP-1 RA	4	663	6.03
Multivariable and IPTW-weighted Cox models for chronic kidney disease progression			
Unadjusted log-rank	HR	95% CI	p-value
Overall group comparison	-	-	0.30
Variable - multivariable Cox model	HR	95% CI	p-value
SGLT2i versus SGLT2i + GLP-1 RA	1.34	0.31-5.88	0.698
GLP-1 RA versus SGLT2i + GLP-1 RA	3.14	0.87-11.37	0.698
IPTW-weighted Cox model			
SGLT2i versus SGLT2i + GLP-1 RA	1.56	0.34-7.21	0.572
GLP-1 RA versus SGLT2i + GLP-1 RA	2.89	0.79-10.57	0.109

Combined therapy resulted in a 1.0% HF outcome rate, compared to 5.1% for SGLT2is and 3.3% for GLP1-RAs alone; however, this difference was not statistically significant (p>0.05). Combining therapy resulted in a 4.0% worsening CKD event rate, compared to 5.1% for SGLT2i and 9.8% for GLP1-RA monotherapy. However, these differences were not statistically significant (p>0.05). No statistically significant differences in side effects were observed between combined GLP-1 RA and SGLT2i therapy and either agent alone, as shown in Table 6.

**Table 6:** Side effects analysis The chi-square test was used to compare categorical variables. GLP-1 RA: glucagon-like peptide-1 receptor agonist, SGLT2i: sodium-glucose cotransporter 2 inhibitors

Adverse effects that led to discontinuation (events/number at risk (%))	SGLT2i (n=78)	GLP-1 RA (n=61)	SGLT2i + GLP-1 RA (n=99)	SGLT2i + GLP-1 RA versus SGLT2i (chi-square value - χ²)	SGLT2i + GLP-1 RA versus SGLT2i (p-value)	SGLT2i + GLP-1 RA versus GLP-1 RA (chi-square value - χ²)	SGLT2i + GLP-1 RA versus GLP-1 RA (p-value)
Nausea/vomits	2 (2.6)	3 (4.9)	2 (2.0)	χ²=0.064	p=0.801	χ²=1.072	p=0.300
Hypoglycemia	0 (0.0)	1 (1.6)	0 (0.0)	-	-	χ²=1.028	p=0.310
Urinary tract infection	2 (2.6)	0 (0.0)	0 (0.0)	χ²=1.470	p=0.225	-	-

## Discussion

Using data from patients followed in our clinic, we verified that patients treated with both GLP-1 RAs and SGLT2is had lower observed rates of MACE, CKD progression, and HF than those treated with either drug alone, although these differences were not statistically significant, likely due to the sample size and the low incidence of clinical events. We may also consider the impact of the lower use of angiotensin-converting enzyme inhibitors/angiotensin II receptor antagonists in the group treated with GLP-1 RAs and SGLT2is.

The cardiovascular and renal protection associated with dual therapy may reflect additive effects via distinct mechanisms of action. GLP-1 RAs mimic glucagon-like peptide-1 and act at multiple sites. In the pancreas, they increase glucose-dependent insulin secretion and reduce glucagon release; in the stomach, they slow gastric emptying; and in the hypothalamus, they act as an anorexigenic signal [1,2]. These effects increase satiety, reduce food intake, and lead to weight reduction [1]. GLP-1 RAs may also improve cardiovascular and renal outcomes by reducing inflammation and oxidative stress, thereby decreasing albuminuria and glomerular hyperfiltration, and improving endothelial function [3-5].

SGLT2i inhibits sodium-glucose cotransporter 2 in the kidneys, reducing the renal threshold for glucose excretion, increasing glycosuria, and leading to calorie loss [6,7]. They also reduce blood pressure and improve arterial elasticity through osmotic diuresis and natriuresis, decreasing cardiac preload and afterload and, lastly, lowering HF risk [8,9]. The kidneys’ protection is due to reduced sodium reabsorption in the proximal tubule, which lessens hyperfiltration and lowers intraglomerular pressure [10]. Short-term, small-scale trials suggest that combining GLP-1 RAs with SGLT2is improves glycemia, blood pressure, body weight, and albuminuria more than using either agent alone [11,12].

Combining a GLP-1 RA with an SGLT2i results in greater reductions in body weight, HbA1c, and blood pressure than an SGLT2i alone, providing significant cardiovascular benefits [13,14]. In patients with T2D and established atherosclerotic cardiovascular disease (ASCVD) or multiple cardiovascular risk factors, the American Diabetes Association recommends this dual therapy when HbA1c goals are not achieved with either SGLT2i or GLP-1 RA alone [15].

Robust evidence supports the use of GLP-1 RAs and SGLT2is in patients with cardiovascular disease [16-22]. Across several randomized controlled trials, such as CANVAS, CREDENCE, DAPA-CKD, DAPA-HF, DECLARE-TIMI 58, EMPA-KIDNEY, EMPA-REG, and EMPEROR, SGLT2is consistently reduced MACE, HF hospitalizations, and progression of CKD [23]. Similarly, GLP-1 RAs have demonstrated cardiovascular benefits in the LEADER, REWIND, SELECT, and SUSTAIN-6 trials, as well as renoprotective effects in the FLOW trial. Moreover, a recent review found that patients on dual therapy had fewer cardiovascular events than those on control or placebo [24].

We did not find any published randomized trial evaluating the cardiorenal outcomes associated with combined GLP-1 RA and SGLT2i therapy. Nevertheless, the PRECIDENTD trial (ClinicalTrials.gov ID: NCT05390892) is underway to assess whether combination therapy is superior to GLP-1 RA or SGLT2i monotherapy with respect to cardiovascular and renal benefits in patients with T2D and either established ASCVD or high risk for ASCVD.

This study has several strengths. We evaluated a real-world cohort with four-year follow-up, including both cardiovascular and renal outcomes, which have a major impact on the morbidity and mortality of people with T2D. We also had detailed data on patients’ adherence to treatment regimens, as well as complete access to reports and hospital admissions for cardiovascular events. The study reflects a clinical practice population.

This study has several limitations. First, its retrospective observational design precludes causal inference and is subject to residual confounding despite multivariable adjustment and IPTW. Although IPTW substantially improved baseline covariate balance, small residual imbalances remained for variables such as age and BMI.

Second, because this was a retrospective study with a fixed sample size, no a priori sample size calculation was performed. The relatively small cohort and the low number of cardiovascular and renal outcome events limited statistical power, particularly for heart failure hospitalization and cardiovascular mortality. Consequently, hazard ratio estimates were associated with wide confidence intervals and should be interpreted cautiously. Consequently, the absence of statistically significant differences should not be interpreted as evidence of equivalence between treatment groups.

Third, the low number of events limited the stability of the multivariable Cox regression models. Although conventional multivariable analyses suggested lower risks in the combination therapy group for some outcomes, these findings were attenuated and no longer statistically significant after IPTW adjustment, indicating that the results should be interpreted as exploratory rather than confirmatory.

Finally, the relatively short follow-up period and the low incidence of clinical events may have reduced the ability to detect differences between treatment strategies, particularly for outcomes that develop over longer periods, such as cardiovascular events.

## Conclusions

Combination therapy with a GLP-1 RA and an SGLT2i seems to be associated with lower rates of MACE, hospitalization due to HF, and CKD progression compared to either drug class alone; however, the lack of statistical significance limits the conclusions that can be drawn from the study. Larger randomized controlled trials and laboratory studies are needed to confirm these preliminary observations and further investigate cardiorenal protective mechanisms.
